# Impact on quality of life and safety of sublingual and subcutaneous immunotherapy in children with severe house dust mite and pollen-associated allergic rhinoconjunctivitis

**DOI:** 10.1186/s13601-020-00315-0

**Published:** 2020-04-20

**Authors:** Thomas Proctor, Elodie Morrough, Otto Fenske, Sarah Allatt, Stephen M. Hughes, Vibha Sharma, Peter D. Arkwright

**Affiliations:** 1 Department of Paediatric Allergy and Immunology, Royal Manchester Children’s Hospital, Oxford Rd, Manchester, M13 9WL UK; 2 Lydia Becker Institute of Immunology and Inflammation, University of Manchester, Manchester, UK

**Keywords:** Children, Allergic rhinoconjunctivitis, Immunotherapy, Sublingual, Subcutaneous, Pollen, House dust mite, Quality of life

## Abstract

**Background:**

Pollen and house dust mite (HDM) subcutaneous immunotherapy (SLIT) and pollen subcutaneous immunotherapy (SCIT) are effective therapies for children with allergic rhinoconjunctivitis (AR). There are no previous direct comparative studies investigating quality of life (QoL) of all three immunotherapy regimes. The aim of this study was to compare QoL and safety in children receiving these immunotherapies for AR.

**Methods:**

Demographic characteristics, Rhinoconjunctivitis Quality of Life Questionnaire (RQLQ) and Visual Analogue (VAS) scores were assessed in 249 children undergoing HDM and pollen immunotherapy at a UK specialist paediatric centre between 2007 and 2019.

**Results:**

All three immunotherapy regimes led to a > 50% improvement in QoL and VAS after 3 years of therapy, with significant improvements by the end of the first year (p < 0.05) and further improvements between 1 and 3 years (p < 0.05). Age, gender, ethnicity and route of administration had no significant bearing on efficacy. Older, polysensitised children and those receiving HDM SLIT were all more likely to discontinue their treatment (all with p < 0.05). The only patient to suffer from anaphylaxis requiring intramuscular adrenaline, and 80% experiencing exacerbations of their asthma had received pollen SCIT.

**Conclusions:**

Pollen SCIT and pollen and HDM SLIT all lead to significant improvements in QoL. The risk of anaphylaxis is low, but SCIT is associates with a 1 in 5 chance of asthma flares in the days after its administration. Discontinuation of therapy is more frequent in older, polysensitised children, and those undergoing HDM immunotherapy.

## Introduction

Allergic rhinitis (AR) is common in both adults and children, with reported prevalence in the UK of up to 40% in population surveys and 11% in general practice [1]. AR is associated with asthma, and impairs patients’ physical and mental health, school exam performance, and quality of life (QoL) [2, 3]. It is estimated that 60 million US citizens suffer from AR. In 2006, the estimated cost of lost productivity due to AR was $593 per employee per year, exceeding that of stress ($518), depression ($273), and arthritis/rheumatism ($269) [4]. As the incidence of childhood AR is continuing to rise, the impact of disease modifying therapies on QoL deserves further evaluation [5].

With its origins dating back to 1911, subcutaneous immunotherapy (SCIT) involves the injection of allergen extract by trained clinicians. Sublingual immunotherapy (SLIT) was developed and first accepted by the World Health Organisation as a treatment modality for pollen allergy in 1998. The benefit of SLIT was that it could be self-administered and thus only requiring clinic attendance for initial training [6]. Ultra-short course SCIT has since been developed, reducing the number of injections and therefore day-case admissions necessary to achieve desensitisation from 90 to 12 over 3 years [7]. More recently house dust mite (HDM) SLIT has become available, allowing a second common aeroallergen to be treated without the need for injections [8, 9].

The efficacy of SCIT and SLIT pollen, and SLIT HDM are already well established in children [10, 11]. Although meta-analyses suggest that SCIT and SLIT for pollen immunotherapy have similar efficacies [12–16], no studies have directly comparing the relative impact of pollen SCIT and SLIT, and HDM SLIT on QoL in children or adults. It is this gap in our knowledge that the present study aimed to address. This large single-centre survey studied children receiving pollen SCIT, pollen SLIT and HDM SLIT over a 12-year period in order to determine the relative improvement in QoL, safety and withdrawal rates of these therapeutic options.

## Methods

### Study populations

Data were collected retrospectively from 249 children receiving SCIT and SLIT for pollen, and SLIT for house dust mite allergy at the Department of Paediatric Allergy and Immunology, Royal Manchester Children’s Hospital, Manchester, United Kingdom between 2007 and 2019. After referral to this specialist centre for AR, children’s disease severity was recorded, and the impact on their QoL was assessed using the scoring systems described below. Sensitisation to grass and tree pollens, as well as house dust mite (*Der p* and *Der f*) was determined by skin prick testing (Stallergenes Greer, London, UK) and/or specific IgE levels (ImmunoCAP, Uppsala, Sweden). Children were considered for AIT if they were sensitised to one or more of these allergens and their AR remained poorly controlled despite standard therapy (four times the standard dose of a second or third generation antihistamine given orally, with addition of regular corticosteroid nasal spray and olopatadine eye drops in older children able to tolerate these treatments) during the previous pollen season, or the previous 3 months in patients with HDM allergy. Patients were excluded if their symptoms were well controlled on standard therapy, if they were unable to comprehend or comply with regular standard therapy, or if they had symptomatic allergy to pet dander and kept a pet at home. Asthma was brought under control before commencing AIT. Patients who began AIT in other centres were also excluded. After explaining the risks and benefits of AIT, formal written consent was obtained from all children before commencing IT. This survey was done with the agreement and support of our Hospital Audit Department (Audits 8179, 8180, 8180) and as such was exempt from ethics committee submission.

### Immunotherapy

Children with pollen allergy were offered the choice of SLIT or SCIT, while those with HDM allergy were only offered SLIT. In patients with pollen allergy, the final decision between SLIT or SCIT was left to parental preference. Grass, tree, or grass and tree Pollinex quattro SCIT (Allergy Therapeutics, London, UK) were administered pre-seasonally as four injections at weekly intervals for 3 years in the day-case unit. Grass pollen (Grazax^®^ 75,000 SQ-T units once a day, ALK-Abello, Reading, UK), or HDM SLIT (Oralvac^®^, Der p/Der f 50/50, Allergy therapeutics, Worthing, UK)) were given as daily sublingual tablets or drops, the first of which was given in the day-case unit and the rest self-administered at home for 3 years by the patient or their carer, with quarterly clinic appointments to assess compliance and response to treatment. Patients were labelled as having completed the course of therapy after 3 years of treatment and final reassessment in clinic.

### Data collection

Data were gathered via Chameleon electronic health records software and physical notes. Data were anonymised for the purposes of analysis. Clinical severity was measured using two QoL scores completed at each hospital appointment. Completion of QoL scores are a routine part of assessments of children undergoing AIT at our allergy service. The first was the Rhinoconjunctivitis Quality of Life Questionnaire (RQLQ) score developed by Juniper et al. in 1994 [17]. Scores ranged from zero (no symptoms) to 150 (maximum score). Questions covered nasal and eye symptoms, impact on physical and emotional wellbeing, and activities. The RQLQ was developed for children aged 12 to 17 years old. In our study, in children younger than 12 years old who were unable to answer the questions themselves, the questionnaire was completed by the parents. The second QoL score was the Visual analogue score (VAS) with scores ranging from zero (no symptoms) to ten (most severe symptoms) [18].

Side-effects were divided into three categories

1. Non-anaphylactic if patients experienced swelling, urticaria, pruritis or irritation but no respiratory or circulatory symptoms
2. Anaphylactic characterised by rapid-onset of breathing or circulatory problems such as bronchospasm or symptoms of upper airway obstruction requiring intramuscular adrenaline
3. Asthma if the patients had no immediate respiratory symptoms after AIT administration, but required more frequent use of their salbutamol inhaler for their asthma (in the case of SCIT over the following 1–2 days), as assessed at the subsequent visit a week later.

### Statistical analysis

SPSS Statistical Package 25 (IBM, New York, USA) was used to analyse the dataset. Reviewing the skewness and kurtosis showed that some data were not normally distributed and therefore median and interquartile range (IQR) were calculated and non-parametric statistics used to compare the groups. Chi squared analysis was used for discrete variables such as gender and race, whilst Mann–Whitney U Test was used for continuous variables. Binary logistic regression analysis and Cox Regression were used to determine the relative risk (RR) and 95% confidence interval (95% CI) of variables. Results were considered statistically significant if p < 0.05.

## Results

### Demography and baseline severity of allergic rhinoconjunctivitis

**Fig. 1:**
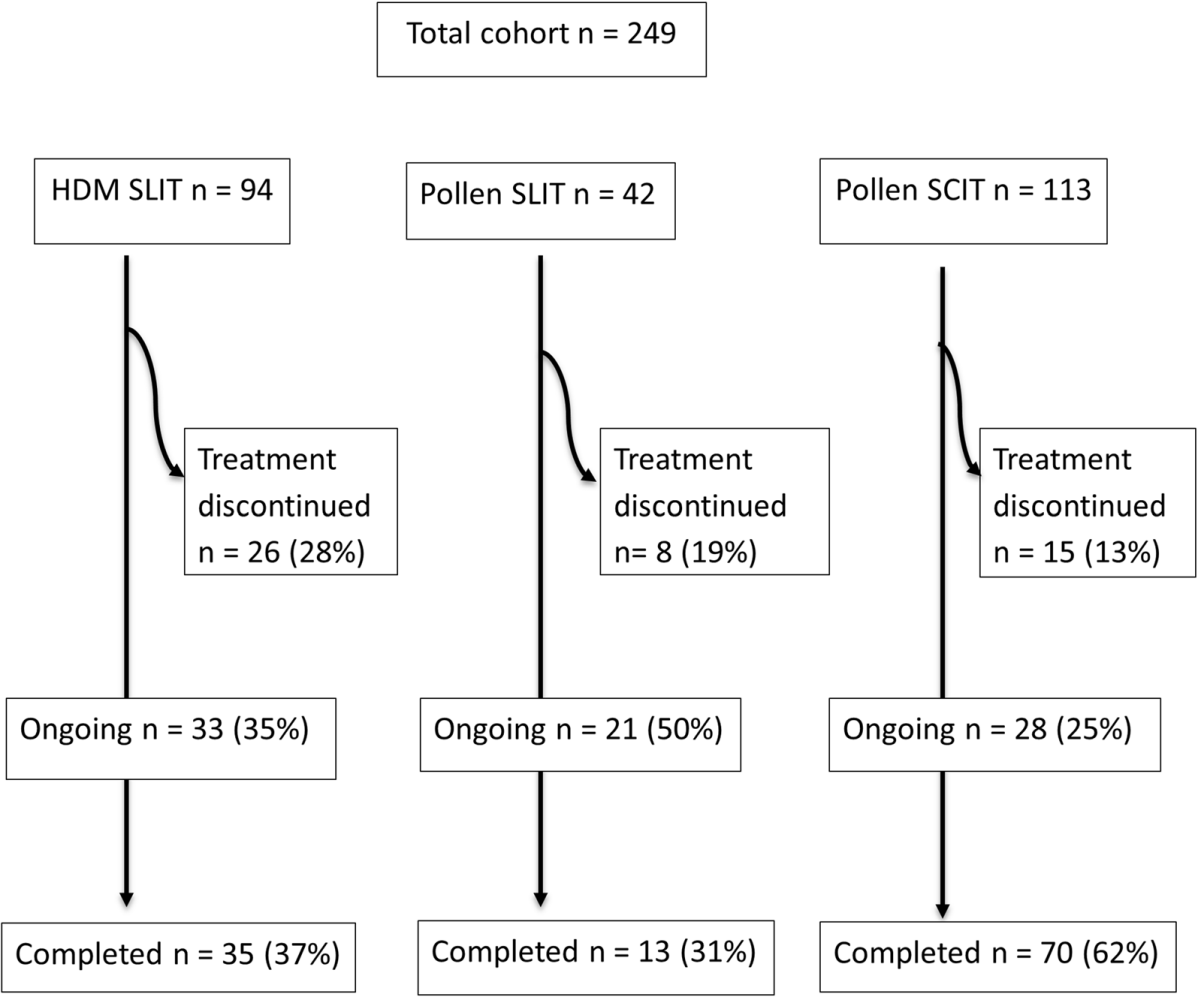
Flow diagram showing the number (percentage) of children treated with HDM SLIT, pollen SLIT or pollen SCIT who completed the full 3 years, discontinued therapy prior to 3 years, or have ongoing therapy

249 children with severe AR started AIT, 94 (38%) HDM SLIT and the remainder pollen AIT (SCIT 113, 45%; SLIT 42, 17%) (Fig. 1). Median age was 13 (range 4–17) years (Table 1). As children aged 4–7 years old only received SLIT, it is not surprising that these children were significantly younger (median (range) age (12 (4–17) years) than those receiving SCIT (14 (8–17) years, p < 0.001). 212 (85%) suffered from both nose and eye disease. 244 (98%) were receiving an oral antihistamine, corticosteroid nasal spray and mast cell stabilising eye drops at the time of recruitment. 174 (70%) were taking regular inhalers for asthma. Sixty (24%) were on regular inhaled corticosteroids, 71 (29%) inhaled steroids combined with a LABA or montelukast, and 15 (6%) were receiving subcutaneous omalizumab.

**Table 1:** Demography and clinical characteristics of total cohort and AIT subgroups

	Total cohort	Pollen SCIT	Pollen SLIT	HDM SLIT	P-value
Number of patients	249	113 (45%)	42 (17%)	94 (38%)	
Patients withdrawn	49 (20%)	15 (13%)	8 (19%)	26 (28%)	0.03
Age (years) at which patients started treatment Median (IQR)	13 (10–15)	15 (12–15)	11 (9–14)	12 (10–15)	< 0.001
Gender					
Male	163 (65%)	77 (68%)	30 (71%)	56 (60%)	0.3
Female	85 (35%)	36 (32%)	12 (29%)	38 (40%)	
Ethnicity					
White European	157 (63%)	84 (74%)	35 (83%)	38 (40%)	< 0.001
Other	92 (37%)	29 (26%)	7 (17%)	56 (60%)	
AR symptoms					
Eyes only	14 (6%)	4 (4%)	7 (17%)	3 (3%)	0.01^b^
Nose only	23 (9%)	7 (6%)	5 (12%)	11 (12%)	
Eyes + nose	212 (85%)	102 (90%)	30 (71%)	80 (85%)	
Allergen sensitisation^a^					
Grass pollen	29 (12%)	17 (16%)	11 (27%)	1 (1%)	< 0.001^b^
Tree pollen	4 (2%)	3 (3%)	1 (3%)	0	
Grass & tree	34 (14%)	27 (26%)	6 (15%)	1 (1%)	
HDM	23 (10%)	1 (1%)	0	22(24%)	
HDM & grass	57 (24%)	13 (13%)	13 (31%)	31 (33%)	
HDM & tree	4 (2%)	0	0	4 (4%)	
HDM, grass & tree	87 (36%)	43 (41%)	10 (24%)	34 (37%)	
Mono-sensitised to AIT					
Yes	98 (40%)	56 (50%)	19 (45%)	23 (25%)	< 0.001
No	151 (60%)	57 (50%)	23 (55%)	71 (75%)	
Medication					
Oral antihistamines	5 (2%)	2 (2%)	1 (2%)	2 (2%)	0.8^b^
OA + adjunct	224 (90%)	99 (88%)	39 (93%)	86 (92%)	
Systemics	20 (8%)	12 (10%)	2 (5%)	6 (6%)	
Other atopic disease					
Asthma	101 (41%)	51 (45%)	13 (31%)	37 (39%)	0.5
Eczema	21 (8%)	10 (9%)	2 (5%)	9 (10%)	
Asthma + Eczema	73 (29%)	31 (27%)	6 (14%)	36 (38%)	
Baseline RQLQ Median (IQR)	65 (38–89)	77 (42–98)	74 (56–105)	57 (28–80)	< 0.01
Baseline VAS Median (IQR)	6 (4–8)	7 (5–8)	8 (7–9)	6 (3–7)	< 0.001

*AIT* allergy immunotherapy, *AR* allergic rhinoconjunctivitis, *HDM* house dust mite, *IQR* interquartile range, *OA* oral antihistamines, *RQLQ* rhinoconjunctivitis quality of life questionnaire, *SCIT* subcutaneous immunotherapy, *SLIT* sublingual immunotherapy, *SPT* skin prick test, *VAS* visual analogue score

P values are between the three treatment groups using Chi squared Test for nominal and Kruskal–Wallis Test for continuous variables. ^a^Sensitisation based on SPT or ImmunoCAP ^b^Fisher’s Exact test used as data violates conditions for Chi squared Test

Males outnumbered females by two to one. There was also a predominance of non-white European children (92 (37%)) when compared with North West of England census data (11% in 2011) [19]. Non-white European predominance was more pronounced for children receiving HDM AIT (56, 60%) than for those receiving pollen AIT (7, 17%) (p < 0.001).

### Withdrawal from AIT

One in five patients discontinued AIT before completing the full 3 years (Fig. 1, Table 2). Twenty-six (53%) because of non-attendance or non-compliance, 12 (24%) because of side-effects (11/12 were in the HDM SLIT group), five (10%) because of lack of perceived benefit, and six (12%) after being transferred to other centres. Children on HDM AIT were twice as likely to discontinue therapy 26/94 (28%) than those on pollen AIT 23/155 (15%), p = 0.02. The risk of discontinuing therapy were also higher in older children (RR (95% CI) 1.3 (1.1–1.4), and those who were polysensitised (35/96, 36%) than those who were mono-sensitised (14/71, 20%, p = 0.02). Multivariate analysis with AIT type and age as variables, showed that age was a significant co-factor (1.2 (1.0–1.4, p = 0.01). A significant difference was also found between HDM SLIT and pollen SLIT (0.3 (0.1–0.8), p = 0.02), but not between HDM SLIT and pollen SLIT (0.8 (0.3–2.6), p = 0.8), or between pollen SLIT and pollen SCIT (2.6 (0.8–8.5), p = 0.1).

**Table 2:** Demographic and clinical features of total cohort and AIT subgroups in patients who completed or withdrew from the AIT program

	Total cohort			Pollen SCIT			Pollen SLIT			HDM SLIT		
	Completed	Withdrew	P-value	Completed	Withdrew	P-value	Completed	Withdrew	P-value	Completed	Withdrew	P-value
Frequency	118 (71%)	49 (29%)		70 (82%)	15 (18%)		13 (62%)	8 (38%)		35 (49%)	26 (51%)	
Age (years) Median (IQR)	12 (10–14)	14 (12–16)	< 0.001	13 (11–14)	15 (14–16)	< 0.001	11 (10–15)	12 (9–16)	0.5	10 (9–13)	14 (11–16)	0.003
Gender												
Male	86 (73%)	29 (59%)	0.08	55 (79%)	9 (60%)	0.2	9 (69%)	6 (75%)	1.0^a^	22 (63%)	14 (54%)	0.5
Female	32 (27%)	20 (41%)		15 (21%)	6 (40%)		4 (31%)	2 (25%)		13 (37%)	12 (46%)	
Race												
White European	87 (74%)	31 (26%)	0.1	57 (81%)	12 (80%)	0.6^a^	13 (100%)	7 (88%)	0.4^a^	17 (49%)	11 (42%)	0.6
Other	30 (61%)	49 (39%)		13 (19%)	3 (20%)		0 (0%)	1 (12%)		18 (51%)	15 (58%)	
AR symptoms												
Eyes	8 (7%)	3 (6%)	1.0^a^	3 (4%)	1 (7%)	0.8^a^	3 (23%)	1 (12%)	1.0	2 (6%)	1 (4%)	0.9^a^
Nose	11 (9%)	4 (8%)		4 (6%)	0 (0%)		1 (8%)	1 (12%)		6 (17%)	3 (11%)	
Eyes + nose	99 (84%)	42 (86%)		63 (90%)	14 (93%)		9 (69%)	6 (75%)		27 (77%)	22 (85%)	
Medication												
OA only	3 (3%)	0	1.0^a^	1 (1%)	0%	0.3^a^	0 (0%)	0 (0%)	0.4^a^	2 (6%)	0 (0%)	0.6^a^
OA + Adjuncts	107 (90%)	47 (88%)		65 (93%)	12 (80%)		13 (100%)	7 (88%)		29 (83%)	24 (92%)	
Systemics	8 (7%)	6 (12%)		4 (6%)	3 (20%)		0 (0%)	1 (12%)		4 (11%)	2 (8%)	
Other atopic disease												
Asthma	55 (47%)	17 (35%)	0.2	35 (50%)	6 (40%)	0.2	6 (46%)	2 (25%)	1.0^a^	14 (40%)	9 (35%)	1.0^a^
AD	11 (9%)	3 (6%)		7 (10%)	1 (7%)		0 (0%)	0 (0%)		4 (11%)	2 (8%)	
Asthma + AD	29 (25%)	17 (35%)		12 (17%)	6 (40%)		2 (15%)	1 (12%)		15 (43%)	10 (38%)118	
Mono-sensitised to AIT												
Yes	57 (48%)	14 (29%)	0.02	43 (61%)	4 (27%)	0.03	6 (46%)	3 (38%)	1^a^	8 (23%)	7 (27%)	0.7
No	61 (52%)	35 (71%)		27 (39%)	11 (73%)		7 (54%)	5 (62%)		27 (77%)	19 (73%)	
Median baseline RQLQ (IQR)	65 (36–89)	59 (38–85)	0.5	77 (38–97)	75 (41–89)	0.5	56 (45–115)	80 (80–80)	0.9	51 (33–79)	15 (43%)	0.3
Median baseline VAS (IQR)	7 (5–8)	6 (4–8)	0.8	7 (4–8)	7 (4–9)	0.9	8 (6–9)	7 (6–7)	0.4	7 (5–7)	6 (2–7)	0.9

*AIT* allergy immunotherapy, *AR* allergic rhinoconjunctivitis, *HDM* house dust mite, *IQR* interquartile range, *OA* oral antihistamines, *RQLQ* rhinoconjunctivitis quality of life questionnaire, *SCIT* subcutaneous immunotherapy, *SLIT* sublingual immunotherapy, *VAS* visual analogue score

P-values between completed and discontinued subgroups using Chi squared Test for nominal and Mann–Whitney U Test for continuous variables. ^a^Fisher’s Exact test used as data violates conditions for Chi squared Test

### Clinical outcomes

**Fig. 2:**
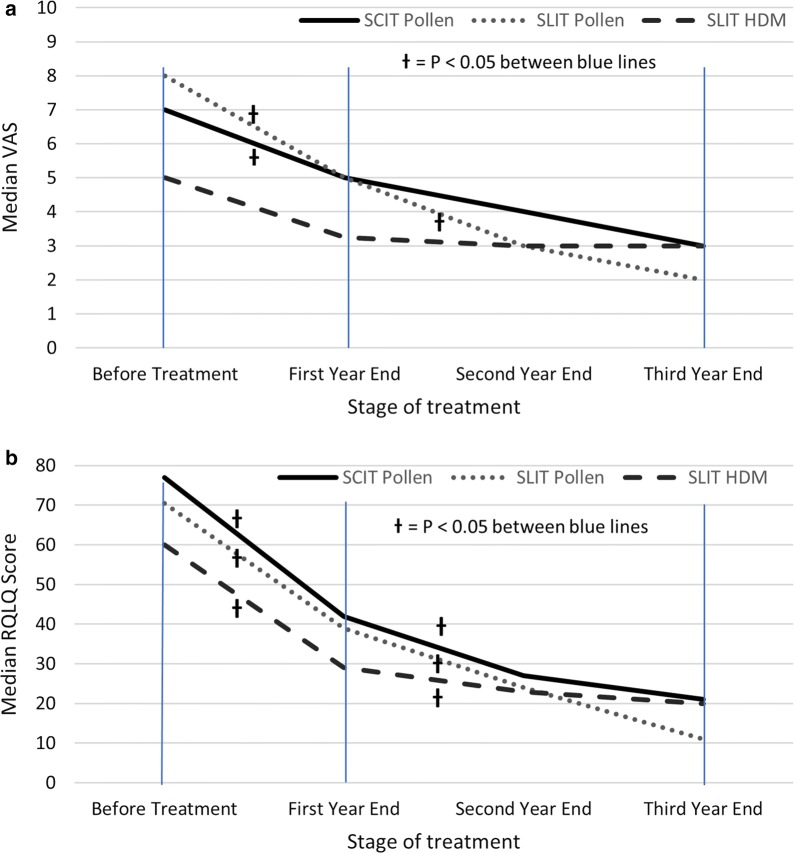
Changes in median Visual analogue and RQLQ scores with AIT. **a** Visual Analogue Score, **b** RQLQ score at baseline and annual intervals. Excludes patients who have discontinued. †P < 0.05 for Wilcoxon signed rank test between baseline and 1 year, or between 1- and 3-years scores

All AIT regimes were associated with significant improvement in QoL scores (Fig. 2). RQLQ improved by > 50% compared to baseline in all groups. Significantly better QoL was apparent by the end of the first year of therapy (41–47% for RQLQ; 29–32% for VAS). Further improvement in RQLQ scores were observed between year one and year three (36–72%), with more variable improvement in VAS. These trends were not affected by age, gender, ethnicity, route of administration, degree of sensitisation, or after excluding patients that withdrew from therapy (data not shown).

### Adverse events

**Fig. 3:**
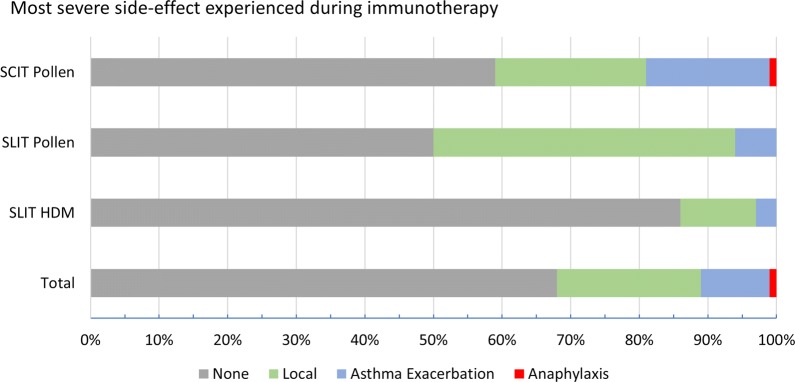
Adverse events experienced by patients in the total cohort and AIT subgroups

221/249 (89%) patients either had no side-effects 169 (68%), or only local reactions 52 (21%) (Fig. 3). Only one of 249 (< 1%) patients experienced anaphylaxis requiring intramuscular adrenaline during the 12 years of AIT at our centre. A 13-year-old boy on Step 3 of asthma therapy (British Thoracic Society/Scottish Intercollegiate Guidelines Network) [20] developed facial oedema and bronchospasm requiring intramuscular adrenaline 10 min after receiving an injection in the third year of pollen SCIT therapy. Twenty-five (10%) children developed asthma exacerbations requiring more frequent use of their salbutamol inhaler. This was particularly noticeable in the 1–2 days following pollen SCIT (20/113, 18%), compared with those receiving daily pollen or HDM SLIT (5/136, 4%) (p < 0.001). Frequency of asthma exacerbations were just as common in children using regular inhaled corticosteroids (16/143, 11%) as in those on no inhalers or just using intermittent inhaled salbutamol (9/76, 11%).

Children receiving pollen SLIT were more likely to experience local oral side-effects (15/42, 36%), compared with HDM SLIT (10/94, 11%) or pollen SCIT (25/113, 22%), p < 0.001. 10/26 (38%) of HDM SLIT patients who discontinued therapy experienced side-effects (often flares of atopic dermatitis), compared to 4/68 (4%) who continued with their therapy (p < 0.001).

## Discussion

This is the first study to directly compare the QoL, adherence and safety of pollen SCIT, pollen SLIT and HDM SLIT in children. All therapies showed a > 50% improvement in RQLQ over the 3 years of treatment, and the efficacy of no therapy was significantly better than any other. Significant improvements in RQLQ were seen in all groups after 1 year, with further significant improvements from year one to year three. The trends using VAS were similar but less robust. Discontinuation rates were significantly higher in older, polysensitised children. Children treated with HDM SLIT (28%) were twice as likely to discontinue therapy than those treated with pollen AIT (14%). Multivariate logistic analysis suggested that both route of allergen administration and allergen type were jointly important. A larger cohort size may help to further dissect out the relative important of these two factors.

All three therapies had a good safely record, although there was a significant increase in asthma flares in the SCIT compared with the SLIT group, which should be considered in deciding on route of AIT. There was an overall preponderance of boys, and non-white Europeans (mainly children with ancestors from the Indian subcontinent) receiving HDM SLIT. Despite these demographic trends, neither gender not ethnicity influenced clinical outcome.

Direct comparison with the published literature is not possible, as there are no previously published reports comparing pollen SLIT and HDM SLIT, or pollen SCIT and HDM SLIT. However, reviewing randomised placebo-controlled trials published in children over the last 10 years, our discontinuation rate for pollen SCIT (13%) and SLIT (19%) were within the range of these studies (SCIT 6–13%; SLIT 6–25%) [21–26]. In contrast, our discontinuation rate of HDM SLIT patients (28%) was above the reported range (0–16%) [27–32]. All but one published trial showed statistically significant improvement in symptom scores compared to the placebo, in keeping with the significant improvement in QoL we found across all three AIT groups. Frequency of side-effects events for pollen (6%) and HDM (3%) SLIT also fell within the ranges of previous studies (pollen SLIT 1–15%, HDM 0–13%). The only patients to develop anaphylaxis requiring intramuscular adrenaline and 20/25 (80%) of children who developed exacerbations of their asthma had received SCIT rather than SLIT. Although the risk of anaphylaxis with SCIT is well recognised [33], more insidious exacerbations of asthma in the following days is less well recognised and should be taken into account when deciding on AIT route.

### Study strengths and limitations

The limitations of our study include the relatively small cohort size and the lack of a placebo control group. However, the advantage of this single centre study is that it avoids the potential cofounding effects of variations in patient demographics, clinical approaches to recruitment and data recording.

Areas for future improvement would be increasing compliance with HDM SLIT therapy. Furthermore, the reasons for the over-representation of males and in the HDM group, children from non-white European backgrounds deserves further investigation as it may well provide clues as to the reasons for high prevalence rates, where at present, there is little or no published literature.

## Conclusions

Children with severe AR receiving pollen SCIT and SLIT, and HDM SLIT have similar and significant improvements in QoL. QoL improves after 1 year of therapy and is even greater by the end of 3 years. Anaphylaxis with AIT is uncommon, but asthma exacerbations during the 1–2 days following pollen SCIT occurred in nearly 1 in 5 children, highlighting the importance of optimising asthma management in patients undergoing this form of AIT. Discontinuation of the therapy was more frequent in older, polysensitised children, and those undergoing HDM than with pollen AIT. Further studies are needed to help understand the reasons for the preponderance of males and non-white European children undergoing AIT.

## Data Availability

The data that support the findings of this study are available from the corresponding author upon reasonable request.

## Abbreviations

AIT: Allergy immunotherapy
AR: Allergic rhinoconjunctivitis
CI: Confidence interval
Der f: *Dermatophagoides farinae*
Der p: *Dermatophagoides pteronissinus*
HDM: House dust mite
IQR: Interquartile range
LABA: Long-acting beta-agonist
OA: Oral antihistamine
QoL: Quality of life
RQLQ: Rhinoconjunctivitis Quality of Life Questionnaire
RR: Relative risk
SCIT: Subcutaneous immunotherapy
SLIT: Sublingual immunotherapy
SPT: Skin prick test
VAS: Visual analogue score

## References

[1] Pols DH, Wartna JB, Moed H (2016). Atopic dermatitis, asthma and allergic rhinitis in general practice and the open population: a systematic review. Scand J Prim Health Care.

[2] Canonica G, Mullol J, Pradalier A (2008). Patient perceptions of allergic rhinitis and quality of life: findings from a survey conducted in europe and the United States. World Allergy Organ J..

[3] Walker S, Khan-Wasti S, Fletcher M, Cullinan P, Harris J, Sheikh A (2007). Seasonal allergic rhinitis is associated with a detrimental effect on examination performance in United Kingdom teenagers: case-control study. J Allergy Clin Immunol..

[4] Linneberg A, Dam Petersen D, Hahn-Pedersen J, Hammerby E, Serup-Hasen N, Boxall N (2016). Burden of allergic respiratory disease: a systematic review. Clin Mol Allergy.

[5] Simpson CR, Newton J, Hippisley-Cox J (2008). Incidence and prevalence of multiple allergic disorders recorded in a national primary care database. J R Soc Med.

[6] Canonica GW, Bousquet J, Casale T (2009). Sub-Lingual Immunotherapy: world allergy organization position paper 2009. World Allergy Organ J..

[7] Rosewich M, Lee D, Zielen S (2013). Pollinex Quattro: an innovative four injections immunotherapy in allergic rhinitis. Hum Vaccin Immunother..

[8] Cho SW, Han DH, Kim JM (2018). House dust mite sublingual immunotherapy in allergic rhinitis. Immunotherapy.

[9] Nolte H, Maloney J (2018). The global development and clinical efficacy of sublingual tablet immunotherapy for allergic diseases. Allergol Int..

[10] Pajno GB, Barberio G, De Luca F (2001). Prevention of new sensitizations in asthmatic children monosensitized to house dust mite by specific immunotherapy. A six-year follow-up study. Clin Exp Allergy.

[11] Durham SR, Walker SM, Varga E-M (1999). Long-term clinical efficacy of grass-pollen immunotherapy. N Engl J Med.

[12] Meadows A, Kaambwa B, Novielli N (2013). A systematic review and economic evaluation of subcutaneous and sublingual allergen immunotherapy in adults and children with seasonal allergic rhinitis. Health Technol Assess.

[13] Sayedelahl MA, Nasr NN, Akr MH (2015). Subcutaneous versus sublingual immunotherapy for allergic rhinitis therapy: which Is Superior. Int J Immunol..

[14] Khinchi MS, Poulsen LK, Carat F (2004). Clinical efficacy of sublingual and subcutaneous birch pollen allergen-specific immunotherapy: a randomized, placebo-controlled, double-blind, double-dummy study. Allergy.

[15] Ibrahim BM, Abdel-Latif RS (2018). Comparison between sublingual immunotherapy and subcutaneous immunotherapy in the treatment of pollen-induced vernal keratoconjunctivitis in children. Delta J Ophthalmol..

[16] Yukselen A, Kendirli SG, Yilmaz M (2012). Effect of one-year subcutaneous and sublingual immunotherapy on clinical and laboratory parameters in children with rhinitis and asthma: a randomized, placebo-controlled, double-blind, double-dummy study. Int Arch Allergy Immunol.

[17] Juniper EF, Guyatt GH (1991). Development and testing of a new measure of health status for clinical trials in rhinoconjunctivitis. Clin Exp Allergy.

[18] Klimek L, Bergmann K-C, Biedermann T (2017). Visual analogue scales (VAS): measuring instruments for the documentation of symptoms and therapy monitoring in cases of allergic rhinitis in everyday health care: Position Paper of the German Society of Allergology (AeDA) and the German Society of Allergy and Clinical Immunology (DGAKI), ENT Section, in collaboration with the working group on Clinical Immunology, Allergology and Environmental Medicine of the German Society of Otorhinolaryngology, Head and Neck Surgery (DGHNOKHC). Allergo J Int..

[19] Office for National Statistics. 2011 Census: population estimates for the United Kingdom. UK Census; 2011.

[20] SIGN 158 British guideline on the management of asthma. A national clinical guideline. Scottish Intercollegiate Guidelines Network/British Thoracic Society; July 2019.

[21] Valovirta E, Petersen TH, Piotrowska T (2018). Results from the 5-year SQ grass sublingual immunotherapy tablet asthma prevention (GAP) trial in children with grass pollen allergy. J Allergy Clin Immunol..

[22] Wahn U, Klimek L, Ploszczuk A (2012). High-dose sublingual immunotherapy with single-dose aqueous grass pollen extract in children is effective and safe: a double-blind, placebo-controlled study. J Allergy Clin Immunol..

[23] Ahmadiafshar A, Maarefvand M, Taymourzade B (2012). Efficacy of sublingual swallow immunotherapy in children with rye grass pollen allergic rhinitis: a double-blind placebo-controlled study. Iran J Allergy Asthma Immunol..

[24] Blaiss M, Maloney J, Nolte H (2011). Efficacy and safety of timothy grass allergy immunotherapy tablets in North American children and adolescents. J Allergy Clin Immunol..

[25] Bufe A, Eberle P, Franke-Beckmann E (2009). Safety and efficacy in children of an SQ-standardized grass allergen tablet for sublingual immunotherapy. J Allergy Clin Immunol..

[26] Möller C, Dreborg S, Ferdousi HA (2002). Pollen immunotherapy reduces the development of asthma in children with seasonal rhinoconjunctivitis (the PAT-study). J Allergy Clin Immunol..

[27] de Bot CMA, Moed H, Berger MY (2012). Sublingual immunotherapy not effective in house dust mite-allergic children in primary care. Pediatr Allergy Immunol.

[28] Shao J, Cui Y, Zheng Y (2014). Efficacy and safety of sublingual immunotherapy in children aged 3–13 years with allergic rhinitis. Am J Rhinol Allergy..

[29] Aydogan M, Eifan AO, Keles S (2013). Sublingual immunotherapy in children with allergic rhinoconjunctivitis mono-sensitized to house-dust-mites: a double-blind-placebo-controlled randomised trial. Respir Med.

[30] Yonekura S, Okamoto Y, Sakurai D (2010). Sublingual Immunotherapy with house dust extract for house dust-mite allergic rhinitis in children. Allergol Int..

[31] Okamoto Y, Fujieda S, Okano M (2019). Efficacy of house dust mite sublingual tablet in the treatment of allergic rhinoconjunctivitis: a randomized trial in a pediatric population. Pediatr Allergy Immunol.

[32] Masuyama K, Okamoto Y, Okamiya K (2018). Efficacy and safety of SQ house dust mite sublingual immunotherapy-tablet in Japanese children. Allergy.

[33] Lim CE, Sison CP, Ponda P (2017). Comparison of pediatric and adult systemic reactions to subcutaneous immunotherapy. J Allergy Clin Immunol Pract.

